# Adsorption of Bisphenol A to a Carbon Nanotube Reduced Its Endocrine Disrupting Effect in Mice Male Offspring

**DOI:** 10.3390/ijms150915981

**Published:** 2014-09-10

**Authors:** Wenwei Wang, Cuijuan Jiang, Ledong Zhu, Nana Liang, Xuejiao Liu, Jianbo Jia, Chengke Zhang, Shumei Zhai, Bin Zhang

**Affiliations:** Key Laboratory of Colloid and Interface Chemistry, Ministry of Education, School of Chemistry and Chemical Engineering, Shandong University, Jinan 250100, China; E-Mails: wangwenwei198808@163.com (W.W.); cjjiang@sdu.edu.cn (C.J.); 2004harrypotter@163.com (L.Z.); liangnanas@gmail.com (N.L.); liuxuejiao26@163.com (X.L.); jiajianbo03@gmail.com (J.J.); chengkezhang@gmail.com (C.Z.); smzhai@sdu.edu.cn (S.Z.)

**Keywords:** carbon nanotubes (CNTs), Bisphenol A (BPA), contaminants adsorption, environmental endocrine disrupting effect, reproductive toxicity

## Abstract

Soluble carbon nanotubes (CNTs) have shown promise as materials for adsorption of environmental contaminants such as Bisphenol A (BPA), due to the high adsorption capacity and strong desorption hysteresis of BPA on CNTs. The adsorption of BPA to CNTs may change the properties of both BPA and CNTs, and induce different toxicity to human and living systems from that of BPA and CNTs alone. Herein, we report that oral exposure of BPA/MWCNT–COOH (carboxylated multi-walled carbon nantubes) adduct to mice during gestation and lactation period decreased the male offspring reproductive toxicity compared with those induced by BPA alone. The adduct decreased malondialdehyde (MDA) level in testis and follicle-stimulating hormone (FSH) in serum, but increased the level of serum testosterone in male offspring in comparison to BPA alone. Our investigations broadened the knowledge of nanotoxicity and provided important information on the safe application of CNTs.

## 1. Introduction

Carbon nanotubes (CNTs) display unique physical and chemical properties that enable their wide applications in the fields of biomedicine, biosensor and environmental science [1,2]. Because of their hollow, layered structure and large specific surface area, CNTs is becoming one of the most commonly used nanomaterials to remove various organic compounds such as dioxin, fluoride, chlorobenzenes and endocrine disruptors [3,4], heavy metal [3,5,6,7,8,9] and biological contaminants, including bacteria, virus and cyanobacterial toxins [10]. The main driving forces acting on environmental pollutant–CNT interactions are non-covalent forces, including hydrophobic interaction, electrostatic forces, hydrogen bonding, van der Waal forces and π–π stacking [11].

Given their strong affinity for environmental pollutants, CNTs’ effects on the fate and accumulation of these pollutants have attracted more and more attention, because the adsorption of pollutants to CNTs may change the properties of both pollutants and CNTs, and induce different toxicity to human and living system from that of pollutants and CNTs alone. Some studies showed that CNTs reduced the bioavailability of organic compounds in environment [12,13,14]. A recent work showed that single-walled nanotubes (SWCNTs) or multi-walled nanotubes (MWCNTs) substantially decreased pyrene bioaccumulation from contaminated soil by earthworms [2], while another study showed that CNT increased the diuron toxicity through locally elevating exposure concentration in the proximity of algal cells [15]. However, according to our knowledge, the effects of CNTs on the fate of environmental pollutants are rarely investigated *in vivo* even though the study of toxicity from nanoparticle alone has been a research focus in recent years, and the investigation on health impact of pollutant–CNTs adducts is quite imperative.

Bisphenol A (BPA) has been widely used for the production of epoxy resin and polycarbonate plastic. As one of the estrogenic endocrine disrupting chemicals, BPA has been detected in the bodies of more than 90 percent of the human population [16]. According to a recent study, the kinetics of BPA metabolism in women, female monkeys, and CD-1 female mice were very similar, suggesting that studies in mice might be relevant for estimating toxic effects of BPA in humans [17]. BPA has long been shown to produce reproductive and embryonic toxicity [18]. It was found that the exposure of BPA disturbed the fetal reproductive system, accompanied with the hormone dyssecretosis and reproductive organ damage. The exposure of BPA affect the luteinizing hormone (LH) and estrous cycle of female offspring, and change the activity of LH, prolactin and intracerebral aromatase of male offspring [19]. From gestational day (GD) 12 to the end of lactation, exposed the maternal mice to BPA by food at a dose of 2.4 μg/kg/day resulted in the decrease of the testosterone contents in male offspring testis [20]. It is reported that the weight of male offspring’s epididymis and seminal vesicle decreased after maternal mice were orally exposed to BPA at 2 μg/kg/day, while the male offspring’s daily sperm production decreased after adjusting BPA dose up to 20 μg/kg/day [21]. When exposed BPA to pregnant Sprague–Dawley rats at 0.1 and 50 mg/kg/day by food, the sperm forming process in male offspring did not show difference. However, the testis weight and the number of sertoli cells increased in these two different dose groups [22].

CNTs have been considered as an excellent adsorbent for BPA [23]. The molecular simulation of BPA showed that each BPA molecule has a unique shape, like a “butterfly”. The specific steric configuration makes BPA tend to interact with CNTs on the curved surface. On the other hand, BPA could enter the grooves and gaps of CNTs’ tube bundle, which leads to a large increase of BPA’s adsorption. π–π accumulation, the main driving force between BPA and CNTs, together with their molecular structure’s coincidence, make CNTs have high adsorption capacity to BPA. In addition, the CNTs/BPA adduct was found to show obvious hysteresis by desorption. However, the reproductive and embryonic effects of BPA/CNT adduct have not been characterized by far. Therefore, in this work, we focus on how carbon nanotubes affects the reproductive toxicity of BPA in male mouse.

Some of recent studies have proved that carboxylated multi-walled nanotube (MWCNT–COOH) exhibited relatively low toxicity *in vitro* and *in vivo*. For example, MWCNT–COOH reduced the toxicity of MWCNT on L02 cells through decreased activation of mitochondrial apoptotic pathway [24]. In another study [25], although both SWCNT-COOH and MWCNT–COOH altered the expression levels of a small portion of proteins in Caco-2/HT29-MTX co-cultures, they were not sufficient to affect the canonical pathways. Thus cytotoxicity, loss of cell integrity and ROS generation were not observed. Considering its good biocompatibility, we selected MWCNT–COOH to adsorb BPA and evaluated the reproductive toxicity of male offspring by repeated oral exposure of BPA/MWCNT–COOH adduct to female mice during gestation and lactation period. The results indicated that the BPA/MWCNT–COOH adduct decreased the reproductive toxicity on male offspring compared with those induced by BPA alone under the same experimental conditions.

## 2. Results

### 2.1. Properties of MWCNT–COOH and BPA/MWCNT–COOH Adduct

The characterization data of MWCNT–COOH and BPA/MWCNT–COOH adduct are summarized in Table 1. The TEM image of MWCNT–COOH and BPA/MWCNT–COOH adduct clearly shows that there were no difference in the morphology, and Zeta potential measurement by electrophoretic light scattering indicated that MWCNT–COOH and BPA/MWCNT–COOH adduct had similar surface electrostatic and electrodynamic properties in water. The result indicated that the absorption of BPA has no effect on the proterties of MWCNT–COOH.

**Table 1:** Characterizations of multi-walled nanotube (MWCNT)–COOH and Bisphenol A (BPA)/MWCNT–COOH adduct.

TEM	MWCNT–COOH	BPA/MWCNT–COOH
	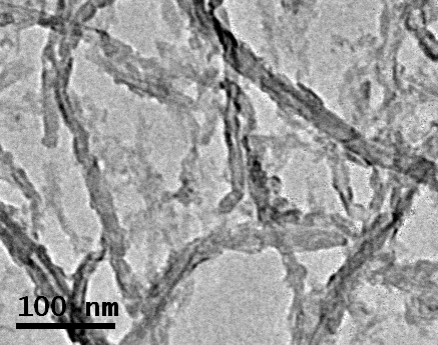	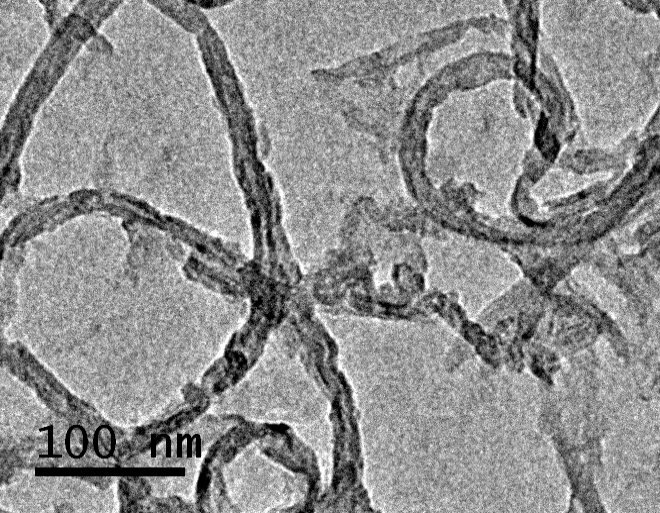
Diameter (nm) inner/outer	~20–30	~20–30
Length distribution (μm)	~0.5–2.0	~0.5–2.0
Zeta potential (mV) in water	−37 ± 1.0	−33.6 ± 0.46
Adsorption (BPA μg/mg MWCNT–COOH)		37 ± 7.5
Desorption/BPA after 24 h		1%

### 2.2. The Adsorption and Desorption of BPA to MWCNT–COOH

**Figure 1:**
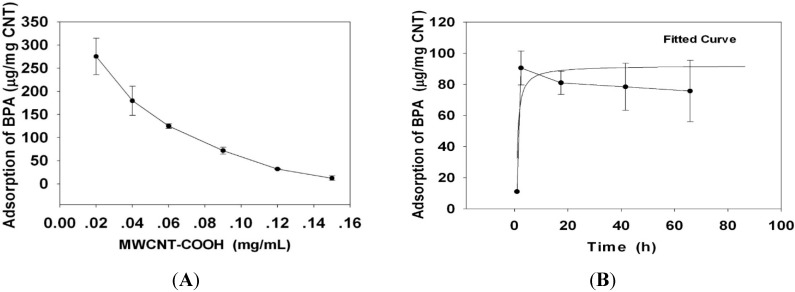
Effect of MWCNT concentration and time on the absorption of BPA. (**A**) Absorption–concentration curve for MWCNT/BPA (Concentration of BPA: 20 μg/mL); (**B**) Absorption-time curve for MWCNT/BPA (Concentration of MWCNT: 0.1 mg/mL).

Based on a recent report, the amount of BPA detected in the real water samples, such as in stream, river and landfill leacheate samples, is usually less than 20 μg/mL [26]. Twenty microgram per milliliter BPA was therefore selected as model concentration in this work. Adsorption curve in Figure 1 showed that the adsorption of BPA to MWCNT–COOH can be completed within a short time, which is a rapid adsorption process according to the studies on the adsorption-concentration relationship of 20 μg/mL BPA to MWCNT–COOH (equilibrate for 24 h) (Figure 1A) and the adsorption-time relationship of BPA to 0.1 mg/mL MWCNT–COOH (Figure 1B).

### 2.3. BPA/MWCNT–COOH Adduct Did not Affect the Maternal Reproduction during Pregnancy

**Figure 2:**
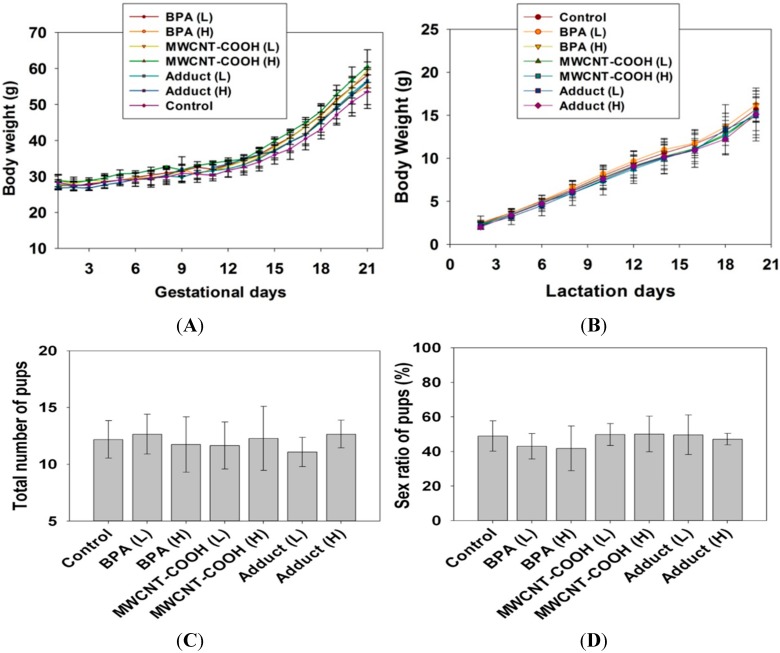
Effect of BPA, MWCNT–COOH or MWCNT–COOH/BPA adduct (all with high (H) and low (L) dose) on the maternal body weight (**A**,**B**), the neonatal numbers (**C**) and sex ratio of offspring (**D**).

According to the adsorption amount of BPA by MWCNT–COOH acquired in the above section, continuous oral exposure of high and low dose of BPA, MWCNT–COOH and their adduct to dams were given during pregnancy and lactation. The changes in dams body weight and dams behaviors during exposure were recorded. The results showed that abnormal maternal behavior was not shown from the beginning of the administration to the end of lactation. As shown in Figure 2A,B, there were no significant differences in body weight between maternal exposure group and control group during both gestational and lactation periods which indicated that maternal exposure of BPA, MWCNT–COOH and their adduct did not make significant effects on dams body weight and behavior.

The number of offspring and proportion of male offspring of each dam was analyzed statistically on gestation day 2. As shown in Figure 2C no significant change in maternal fecundity was found (*p* > 0.05) between exposure group and control group. In addition, the proportion of male mice in each group was about 50%, with no significant change found among these groups (*p* > 0.05) both in low and high dose (Figure 2D). It indicated that oral exposure of BPA, MWCNT–COOH and their adduct at the concentration used in this study did not affect the maternal reproduction during pregnancy.

### 2.4. BPA/MWCNT–COOH Adduct Did not Affect the Body Weight and Organ Index of Male Offspring

**Figure 3:**
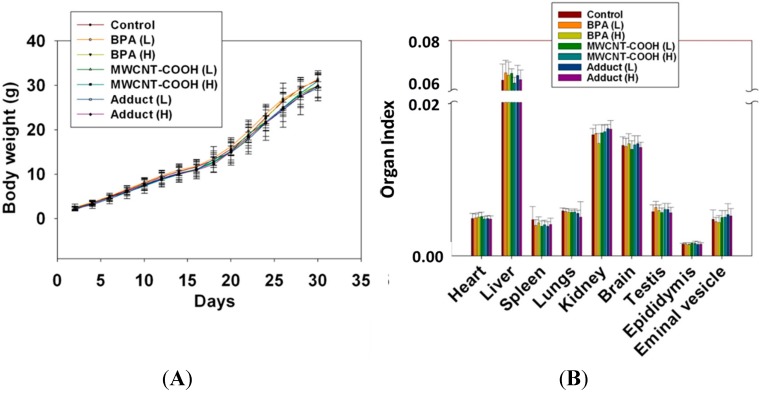
Effects of BPA, MWCNT–COOH and MWCNT–COOH/BPA (all with high (H) and low (L) dose) on the male offsprings body weight and organ index. (**A**) the body weight curve of male offspring; (**B**) the organ index of male offspring including heart, liver, spleen, lungs, kidney, brain, testis, epididymis and seminal vesicle.

The body weight curve showed that body weight of male offspring increased steadily with time (Figure 3A). No significant change was found among these groups (*p* > 0.05). Statistical analysis of male offspring organ index showed that there were no significant changes in organ index of heart, liver, spleen, lung, kidney, brain, testis and epididymis in exposure group compared with control group (Figure 3B). The results indicated that BPA, MWCNT–COOH and BPA/MWCNT–COOH adduct induced no significant toxicity to organs at the concentration used in the study.

### 2.5. The BPA/MWCNT–COOH Adduct Reduced the Effect of BPA on the Male Offspring’s Reproduction System

It is well known that BPA induce reproductive toxicity through the induction of oxidative damage and disturbance of hormones. Therefore, we detected the oxidative damage to testis and the levels of three key reproductive hormones: luteinizing hormone (LH), follicle-stimulating hormone (FSH) and testosterone induced in male offspring by a single oral gavage exposure of BPA, MWCNT–COOH and BPA/MWCNT–COOH adduct to female mice at gestation and lactation period.

**Figure 4:**
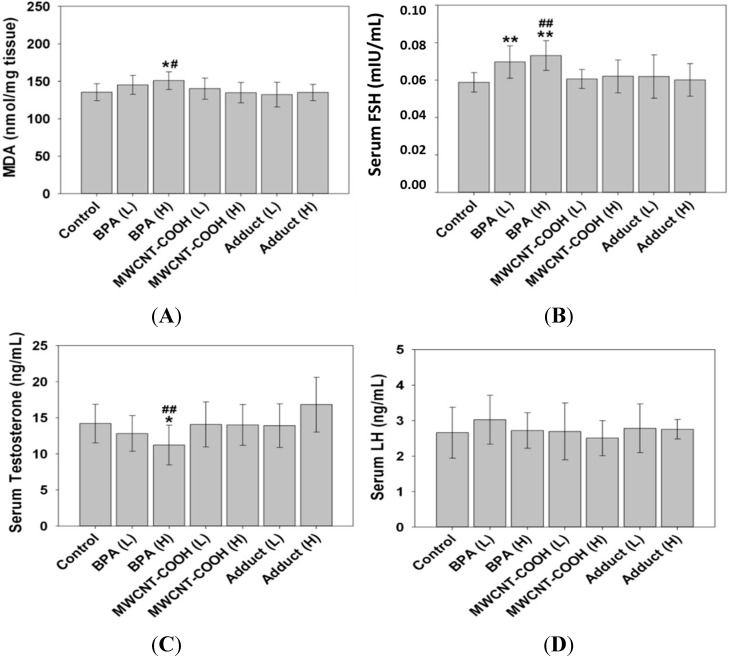
Evaluation of malondialdehyde (MDA) and the serum hormones in male offspring. (**A**) the level of MDA; (**B**) The level of follicle-stimulating hormone (FSH) in serum; (**C**) The level of luteinizing hormone (LH); (**D**) The level of Testosterone. *, **, values derived from *t* tests were significantly different from those of control (* *p* < 0.05 or ** *p* < 0.01); #, ##, values derived from *t* tests were significantly different from those of treated with adduct (H) (# *p* < 0.05 or ## *p* < 0.01).

According to Figure 4A, compared with the control, in the group of BPA high dose (2.4 mg/kg/day), the malondialdehyde (MDA) in testis increased obviously (*p* < 0.05), which means that it would cause lipid peroxidation in male offspring testis by exposure BPA at embryonic and lactation period, which is in accordance with the established reports [27]. However, compared with the BPA group (H), the complex group (H) shows significant reduction in MDA level (*p* < 0.05), which indicates that the level of lipid peroxidation in male offspring testis caused by BPA is reduced when it is adsorbed to nanotubes. In order to further study the effect of BPA/MWCNT–COOH on male mice reproduction, we test the FSH, testosterone, and LH in the offsprings’ serum. The results show that, compared with the control group, the level of FSH increased significantly (*p* < 0.01) in BPA-treated groups at both low and high dose. In contrast, no significant changes (*p* > 0.05) of FSH were observed in other groups. However, the level of FSH is obviously low (*p* < 0.01) in adduct-treated group at high dose when compared to the BPA-treated group at high dose (Figure 4B). Furthermore, high dose BPA reduced the offspring’s testosterone (*p* < 0.05) compared with that of the control group, while the adduct at high dose improved the level of testosterone significantly (*p* < 0.01) compared to that in the BPA-treated groups (Figure 4C). In addition, no significant change of LH was observed in any of the groups (Figure 4D).

## 3. Discussion

Recently, CNTs have been gradually applied for the removal of organic contaminants from wastewater through adsorption process as novel absorbent [28]. Compared with activated carbon and carbon black [13,29,30], CNTs show shorter equilibrium times and higher availability and adsorption capacity due to their highly porous and hollow structure, large specific surface area, and strong interaction between CNTs and pollutant molecules. After the first reports of the bioaccumulation of xenobiotic organic compounds in the presence of carbonaceous nanoparticles in the food chain [31], the potential effects of CNT on the toxicity of organic pollutants has been taken into account. In this work, we assessed whether CNTs affect the reproductive toxicity of BPA by repeated oral exposure of BPA, MWCNT–COOH, and the BPA/MWCNT–COOH adduct to female ICR (CD-1) mice at gestation and lactation period. We investigated their effects on maternal mice and the male offspring. Detection index includes the maternal body weight, the neonatal numbers and sex ratio of offspring, the male offsprings body weight and organ index, the level of MDA in testis and the serum hormones in male offspring.

The results showed that repeated oral exposure of MWCNT–COOH induced relatively low toxicity to maternal reproduction and embryo-fetal toxicity at the concentration used in our study, which was in accordance to previous reports [32,33]. In contrast, BPA exposure induced the testis lipid peroxidation of male offspring, and disturbed the level of FSH and testosterone in serum of male offspring, confirming the environmental estrogen disrupting effects of BPA.

The male offspring testis lipid peroxidation and the change of hormones level (increased LH and decreased testosterone) induced by BPA exposure at gestation and lactation period indicated that BPA can go through the blood-embryo barrier, disturb the axis of offspring’ hypothalamus–hypophysis-testis, and may affect the sperm production in testis as prevously reported [34,35,36]. However, BPA/MWCNT–COOH adduct exposure did not cause significant change of the above indexes indicated that the adsorption of BPA to MWCNT–COOH reduced the toxicity of BPA. In the complex system of mice-BPA/MWCNT–COOH, the strong sorption of BPA on CNT might reduce the reproductive toxicity of BPA through decreasing free BPA. While further research is required to evaluate the toxicity induced by BPA/MWCNT adduct in other system, our work provided important information for the potential application of CNTs as adsorbent for the removal of BPA from the environment.

## 4. Experimental Section

### 4.1. Reagents

Pristine multi-walled carbon nanotubes was purchased from Chengdu Organic Chemicals Co., Ltd., Chinese Academy of Sciences (Chengdu, China). BPA was purchased from Tokyo Chemical Industry Co., Ltd. (TCI) (Shanghai, China). MDA kits were purchased from Nanjing Jiancheng Bioengineering Institute (Nanjing, China). Mouse FSH, Testosterone, and LH ELISA Test Kits were purchased from RapidBio Lab (Calabasas, CA, USA). Other chemicals were purchased from Sigma–Aldrich (St. Louis, MO, USA).

#### 4.1.1. Preparation of MWCNT–COOH

MWCNT–COOH was synthesized according to a previously reported process [37]. Characterizations of MWCNT–COOH were carried out via High-Resolution Transmission Electron Microscope (JEM-2100, JEOL, Tokyo, Japan), Infrared spectroscopy (Nicolet-380, Thermo, Madison, WI, USA), Zeta potential (Zetasizer Nano ZS 90, Malvern, UK) and elemental analysis (Vario EI III, Elemental, Hanau, Germany).

#### 4.1.2. Preparation of BPA/MWCNT–COOH Adduct

Five hundred milliliters of high purity water in round-bottom flask was sterilized at 120 °C for 30 min, and 50 mg BPA was added to obtain a 100 μg/mL BPA solution. The BPA/MWCNT–COOH adduct was prepared by adding 50 mg MWCNT–COOH, which was ultrasonized for 1.5 h for a complete dispersion, to the above solution and then stirred for 24 h.

The obtained solution was concentrated using centrifugal filter (Amicon^®^ Ultra, 10,000 MWCO, Billerica, MA, USA). One hundred and six point eight milliliter complex solution in eight filters (13.35 mL/filter) was centrifuged for 30 min at 5000× *g*. Then the operation was repeated for four times to concentrate all of the adducts. To remove the free BPA completely, the last centrifugation was performed at 5000× *g* for 1.5 h. Then the rest solution was gathered and dispersed in sterile water to get the solution with a final volume of 2.5 mL.

### 4.2. Quantification of BPA Adsorption on MWCNT–COOH

To quantify the BPA adsorbed on MWCNT–COOH, 1.5 mL unconcentrated solution (the solution before concentration) and blank solution (without MWCNT–COOH) were centrifugated at 45,000× *g* for 45 min. One milliliter supernatant and 3.0 mL CH_2_Cl_2_ were mixed in a 7.0 mL tube and were shaken vigorously to extract the BPA in water (All the tubes were boiled for 20 min and washed with CH_2_Cl_2_to remove the inherent BPA). Then the concentration of BPA in CH_2_Cl_2_ was determined by UV–Vis Spectrum at 276 nm (BPA standard solutions: 4.0, 8.0, 12.0, 16.0, 20.0, 24.0, 30.0, 36.0 and 42.0 μg/mL in CH_2_Cl_2_). The amount of BPA on MWCNT–COOH was calculated by the following formula:

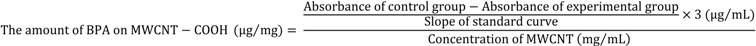



### 4.3. Desorption of BPA from BPA/MWCNT Adduct in Oral Exposure Solution

Four milliliter oral exposure solution was incubated at 37 °C and 1.0 mL solution was centrifugated using centrifugal filter (Amicon^®^ Ultra, 10,000 MWCO, Billerica, MA, USA) every one hour. The concentration of BPA in filtrate at different timepoints were determined by UV–Vis Spectrum at 276 nm (BPA standard solutions: 0, 5.0, 10.0, 20.0, 30.0, 50.0, 100.0, 200.0, 250.0 μg/mL in water). The desorption of BPA from BPA/MWCNT–COOH was calculated by the following Formula:

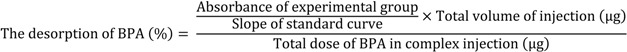



### 4.4. Preparation of the Oral Exposure Solutions for MWCNT and BPA

To prepare the MWCNT oral exposure solution, 50 mg MWCNT–COOH was dissolved in 2.5 mL sterile water and ultrasonized for 1.5 h. To obtain the BPA injection solution, 3.0 mg BPA was dissolved in 10 mL sterile water.

### 4.5. Animal Administration and Sampling

**Figure 5:**
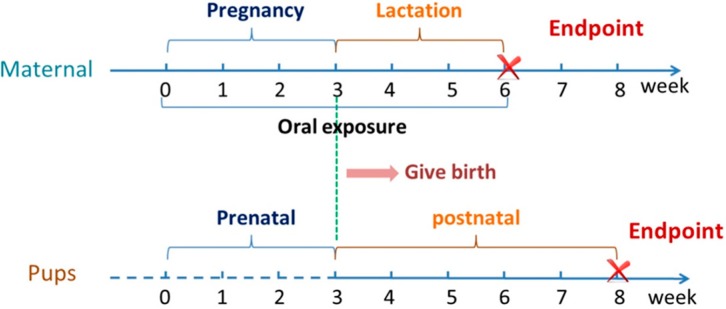
BPA, MWCNT–COOH and BPA/MWCNT–COOH adduct exposure scheme.

ICR (CD-1) mice were purchased from Beijing Vital River Laboratories (Beijing, China). Our experimental protocols complied with the NIH guidelines outlined in the *Guide for the Care and Use of Laboratory Animals* and were approved by a local ethics committee. Animal administration (by a single oral gavage exposure) and sampling were carried out according to the exposure schedule shown in Figure 5. After acclimation for 1 week, 70 female ICR (CD-1) mice (25–29 g) were randomly divided into 7 groups (control, low dose and high dose groups for BPA, MWCNT–COOH and BPA/MWCNT–COOH adduct) with 10 mice per group. These groups were named separately as followed: Control, BPA (L), BPA (H), MWCNT–COOH (L), MWCNT–COOH (H), Adduct (L) and Adduct (H).

Every two female mice were mated with one male mouse (7 weeks old) overnight. The presence of vaginal plaque in the next morning was taken as evidence of mating. This time point was designated as gestation day (GD) 0. The pregnant mice were respectively exposed to water, BPA, MWCNT–COOH or BPA/MWCNT–COOH adduct via oral administration daily from GD 0 to postnatal day (PND) 21. Identification of sex of the F1 generation was carried out on PND 1, and 4–6 male pups of every pregnant mouse were kept with body weight recorded every other day. The F1 generation of mice were separated from the maternal on PND 21 and sacrificed on PND 35. Heart, liver, spleen, lung, kidney, brain, testis, epididymis and seminal vesicles of F1 generation male mice were collected and weighted. The testes were fixed in Bouin solution. The blood was centrifuged at 3000× *g* for 15 min to obtain the serum. The exposure doses for the maternal mice are listed in Table 2.

**Table 2:** The exposure dose for the maternal mice.

Dose	Control	BPA	MWCNT–COOH	Adduct (BPA/MWCNT–COOH)
Low dose	Sterile water	0.8 mg/kg/day	22 mg/kg/day	0.8 mg/22 mg/kg/day
High dose	Sterile water	2.4 mg/kg/day	65 mg/kg/day	2.4 mg/65 mg/kg/day

### 4.6. Reproductive Toxicity of Male Mice

Testes of male mice were collected and weighted. The homogenates prepared from testes were diluted to 10% with PBS and centrifuged at 3000× *g* for 15 min at 4 °C to collect the supernatants for MDA assays. Hormone levels (FSH, Testosterone and LH) in surem of male mice were also detected using a double antibody sandwich ELISA assay according to the manufacturer’s protocol.

### 4.7. Data Analysis

All statistical calculations were carried out using SigmaPlot 12.0 [38]. Results were reported as mean value ± S.D. of multiple determinations. The comparison between the control and experimental groups and differences within experimental groups at different doses were analyzed using *t* test. *p* < 0.05 was considered as an appropriate level of significance.

## 5. Conclusions

In summary, we report that oral exposure of BPA/MWCNT–COOH adduct to mice during gestation and lactation period decreased the male offspring reproductive toxicity compared with those induced by BPA alone. The adduct decreased MDA level in testis and FSH in serum, but increased the level of serum testosterone in male offspring. Our investigations broadened the knowledge of nanotoxicity and provided important information on the safe application of CNTs.
